# Convolutional Recurrent Neural Networks with a Self-Attention Mechanism for Personnel Performance Prediction

**DOI:** 10.3390/e21121227

**Published:** 2019-12-16

**Authors:** Xia Xue, Jun Feng, Yi Gao, Meng Liu, Wenyu Zhang, Xia Sun, Aiqi Zhao, Shouxi Guo

**Affiliations:** 1 School of Information Science and Technology, Northwest University, Xi’an 710127, China; xuexia201606@163.com (X.X.); gaoyi_201908@163.com (Y.G.); liumeng@stumail.nwu.edu.cn (M.L.); zhaoaq2019@163.com (A.Z.); gsxnwu@foxmail.com (S.G.); 2 Maths and Information Technology School, Yuncheng University, Yuncheng 044000, China; 3 State-Province Joint Engineering and Research Center of Advanced Networking and Intelligent Information Services, Northwest University, Xi’an 710127, China; 4 College of Economic and Management, Xi’an University of Posts and Telecommunications, Xi’an 710016, China; zwy201908@163.com; 5 China Aerospace Academy of Systems Science and Engineering, Beijing 100048, China

**Keywords:** personnel performance prediction, self-attention mechanism, convolutional neural networks, long short-term memory, cross-entropy

## Abstract

Personnel performance is important for the high-technology industry to ensure its core competitive advantages are present. Therefore, predicting personnel performance is an important research area in human resource management (HRM). In this paper, to improve prediction performance, we propose a novel framework for personnel performance prediction to help decision-makers to forecast future personnel performance and recruit the best suitable talents. Firstly, a hybrid convolutional recurrent neural network (CRNN) model based on self-attention mechanism is presented, which can automatically learn discriminative features and capture global contextual information from personnel performance data. Moreover, we treat the prediction problem as a classification task. Then, the k-nearest neighbor (KNN) classifier was used to predict personnel performance. The proposed framework is applied to a real case of personnel performance prediction. The experimental results demonstrate that the presented approach achieves significant performance improvement for personnel performance compared to existing methods.

## 1. Introduction

Modern organizations are facing a higher level of increasingly complex competition in global markets. The future survival of organizations depends primarily on personnel performance. Personnel performance, such as capability, conscientiousness, achievement motivation, and other characteristics, plays a critical role in maintaining the competitive advantages of an organization. Accurate personnel performance prediction can help decision makers to recruit and select the most appropriate people for each job. Consequently, personnel performance predicted by computer-aided techniques is an extraordinary challenging research topic.

Many researchers pay close attention to the study on human performance modeling. In the literature, there are many efforts toward forecasting human performance using various methods from psychology, management and data mining areas, such as correlation analysis and rule-based methods [1]. However, rule-based methods have limited performance, because domain experts or professionals need to manually predesign large bodies of rules to explore the meaningful patterns to some extent. Recently, machine-learning-based methods have attracted great attention from some researchers. These methods have also been successfully applied to human performance modeling. Chien et al. [2] presented a data-mining framework using decision tree and association rules for human performance modeling. This study extracted useful patterns and rules between personnel characteristics and work behaviors. A self-regulating clustering algorithm was presented to analyze bank personnel performance in order to improve the performance of human resource management (HRM) [3]. Gobert et al. [4] developed an interactive environment to evaluate students’ scientific research capability via data mining technology. Li et al. [5] proposed an improved KNN algorithm to deal with human performance prediction in a manufacturing system. This method utilized a distance calculation formula based on entropy, a classification rule, and a quantitative description way of human performance. Wang et al. [6] applied a hybrid feature selection method to handle human resource selection, which increased the classification performance. Although many methods have been proposed, depending on the characteristics of good features, these traditional machine-learning-based methods have weak generalization and learning ability. The performance relies too much on prior knowledge of domain designers, which is cumbrous and time-consuming, and heavily affects classification accuracy. However, human performance modeling research is still at an early stage, and there is great potential for further improvements in its performance.

In recent years, deep learning [7,8] and artificial neural networks have exhibited outstanding performance, which have become the state-of-the-art method for many pattern recognition problems. They have been successfully applied in a range of computer tasks, including image classification [9], breast mass classification [10], semantic segmentation [11], object detection and recognition [12,13], emotion recognition [14], language identification [15], agricultural areas [16], drug–drug interaction extraction [17], and so on. However, few works can be found in the field of HRM.

Lately, the rapid development of deep learning has brought new inspiration to HRM tasks. Convolutional neural networks (CNN) [18], which have made a significant breakthrough in feature learning, are a well-known deep learning method. Rather than handcrafted features being extracted, CNN can automatically learn more discriminative features for the current classification task. However, CNN cannot capture the dependency between features which are some distance apart and have semantic relevance. Therefore, we propose a hybrid CRNN model combining CNN with long short-term memory (LSTM) to extract the target features of given personnel performance prediction samples. To better capture global personnel performance information, we introduced self-attention to our CRNN model.

Particularly in the current circumstances, there is generally no prior knowledge about data distribution characteristics. The KNN classifier is the most suitable for processing these data. In addition, KNN can acquire more complicated decision information than the general softmax/sigmoid activation functions at the last layer of self-attention-based CRNN. Self-attention-based CRNN and KNN are completely complementary in terms of feature extraction and decision information. Consequently, we put forward a novel Convolutional Hybrid Recurrent Neural Networks with a Self-Attention Mechanism (called CHRNNA) to make full use of their advantages and be able to make up for their deficiencies.

Recent advances in machine learning suggest that, given sufficient labeled data, there should be an opportunity to construct better prediction models. However, there is no manual labeling of data publicly available. In this work, we created a labeled personnel performance dataset and exploited it using deep learning methods to build an accurate model for personnel performance prediction.

Overall, our contributions in this paper are as follows:

(1) Grounded in psychological theory and management theory of personnel performance, we constructed a high-quality dataset with performance prediction characteristics. This is key to ensuring data quantity and quality;
(2) To the best of our knowledge, this is first time deep learning has been applied to the field of personnel performance prediction, which fills the gap in this field;
(3) Instead of considering each attribute equally, a self-attention mechanism was used to automatically select the informative features of personnel performance. Our proposed CHRNNA framework can be viewed as a universal framework for personnel performance prediction, which has greatly improved classification performance.

The remainder of this paper is structured as follows: The related background is introduced in Section 2; data are characterized in detail, and our proposed method is described thoroughly in Section 3; Section 4 evaluates the effectiveness of our method in a wide range of experiments and presents experimental results as well as analysis; finally, we present general conclusions from our work in Section 5.

## 2. Related Work

The related work can be classified into the following categories, i.e., personnel performance prediction and deep learning research.

### 2.1. Personnel Performance Prediction

In recent years, more and more research results have been devoted to personnel performance prediction. Existing methods used in the investigations are divided into three categories: correlation analysis, rule-based methods, and machine-learning-based methods. The studies of correlation analysis found significant relationships between personal data characteristics, and indicated that a person’s personality traits can be used to predict their future job performance [19,20]. Recently, some researchers have considered the issue from a different perspective. Instead of determining what attributes are best for predicting personnel performance, they analyzed the performance gained by different classifiers. The rule-based methods use manually predefined rules which extract the useful patterns or rules to assist in personnel selection decisions. The limitation is that the classification performance will depend heavily on the ability of domain experts or professionals to design large bodies of rules.

In contrast to rule-based methods, machine-learning-based methods show better generalization. For instance, Cho et al. [21] predicted job performance of insurance sales agents using discriminant analysis and a decision tree. The method obtained a correct classification rate close to 70%, which could help insurance managers tp select quality agents in an insurance industry. Delgado-Gómez et al. [22] developed an expert system using support vector machines to forecast the sale performance of an insurance company. The system reached 5% higher accuracy than the state-of-the-art systems, which could lead to enormously reducing direct or indirect capital expenditure of the companies. Valle et al. [23] forecasted the job performance of sales agents in a call center using a naive Bayesian classifier. Each sample has an attribute vector that characterizes a sale agent. Attributes of each sample are as the input vector of the naive Bayesian model. Through supervised learning, the classifier predicts if the sale agent achieves a minimum production threshold. The classification problem is to judge whether the individual belongs to one class or another. This is a binary problem, and the model classified the instances with an accuracy of 80.60%. Thakur et al. [24] proposed a human performance prediction framework via ensemble-learning technology, which could help decision-makers to recruit a suitable employee in the software industry. Sarker et al. [25] used a hybrid method based on K-Means clustering and a decision tree to forecast staff performance, which could help executives to improve the quality of an organization. However, the biggest challenges of machine-learning-based methods are to choose good features which result in effective learning. Although many traditional classification algorithms have been presented to forecast the performance, the work mentioned above is still in its infancy, and there is great potential for achieving significant performance improvement.

### 2.2. Deep Learning

With the improvement in computing power and the availability of large datasets, deep learning technology has developed rapidly and has achieved unprecedented success in many other tasks. So far, no one has tried to apply deep learning to the personnel performance prediction task of HRM. However, the application of deep learning methods in the literature review is a great point of reference for us.

Deep learning is a family of state-of-the-art techniques. CNN is a popular deep learning technique. Krizhevsky et al. [26] trained a large-scale and deep convolutional neural network to classify images, which achieved the best performance compared to the previous work. CNN has also been used successfully for natural language processing (NLP), involving text classification and sentiment analysis [27,28]. Zhao et al. [29] employed a syntax word embedding to learn the syntactic information of a sentence and fed it into the CNN model to extract drug–drug interactions. This method achieved an F-score of 68.6%, which achieved a better performance than the previous state-of-the-art methods. Ombabi et al. [30] applied deep learning to the Twitter user’s comment classification, employed CNN to extract deep semantic features from the comments, and a support vector machine classifier was used to forecast the final classification. Finally, the topic in which users are interested was obtained, and the proposed method achieved a best accuracy of 97.3% for users’ interest classification. Studies have shown that CNNs have better results than traditional machine learning methods. Although CNNs have been shown to perform well, the classification model which only uses CNNs cannot learn the contextual dependency and structural information.

In addition to CNN models, recurrent neural network (RNN) models have achieved great success in many applications, e.g., object detection [31], video captioning [32], and drug–drug interaction extraction. Huang et al. [33] proposed a two-stage method for improving the performance of drug–drug interaction extraction, which obtained the highest F-score of 69.0%. In the first stage, it identified the positive instances employing a feature-based binary classifier. In the second stage, the LSTM-based classifier was used to classify the positive instances into a specific category. Recently, some researchers have also already successfully applied RNNs to some educational applications, such as student performance prediction, and have shown that they have a better performance than traditional machine learning methods. Deep knowledge tracing (DKT) used recurrent neural networks (e.g., RNN and LSTM) to model a student exercising process to forecast their performances [34]. Furthermore, by exploiting both student exercising records and the text descriptions of exercises, Su et al. [35] proposed an exercise-enhanced recurrent neural network (EERNN) framework for student performance prediction. A bidirectional LSTM was first designed to extract exercise semantic representations from texts. Then, a proposed LSTM architecture was used to trace student states for making final prediction.

Apart from the methods described above, some other works used the combination of CNN and RNN to do classification task. Other classification tasks can also be learned from. Wei et al. [36] employed a network based on CNN and LSTM to automatically identify the urgency of a post and classify the sentimental polarity. This approach used CNN to extract the local contextual feature representations of a post. Then, the post representations were fed into LSTM model to get the long temporal semantic information. The combination of CNN and RNN has also been successfully applied in many other computer tasks, e.g., direction of slip detection [37], multimodal wearable activity recognition [38], and human activity recognition in sensor-rich environments [39]. However, the classification model which only uses the hybrid model based on CNN and LSTM lacks the effective fusion of semantic and structural information. Further, attention-based methods are also popular. Guo et al. [40] used RNN to learn higher-level contextual representations and utilized CNN to gain sentence features for the relation classification task. The hybrid model integrated word-level and sentence-level attention mechanisms, which strengthened critical words and features and improved the performance.

Although deep learning methods have been widely applied in many tasks, for all we know, this is the first work to attempt personnel performance modeling based on deep learning. However, this personnel performance modeling research is still at an early stage, and its prediction performance has much room to improve.

## 3. Methods

**Figure 1:**
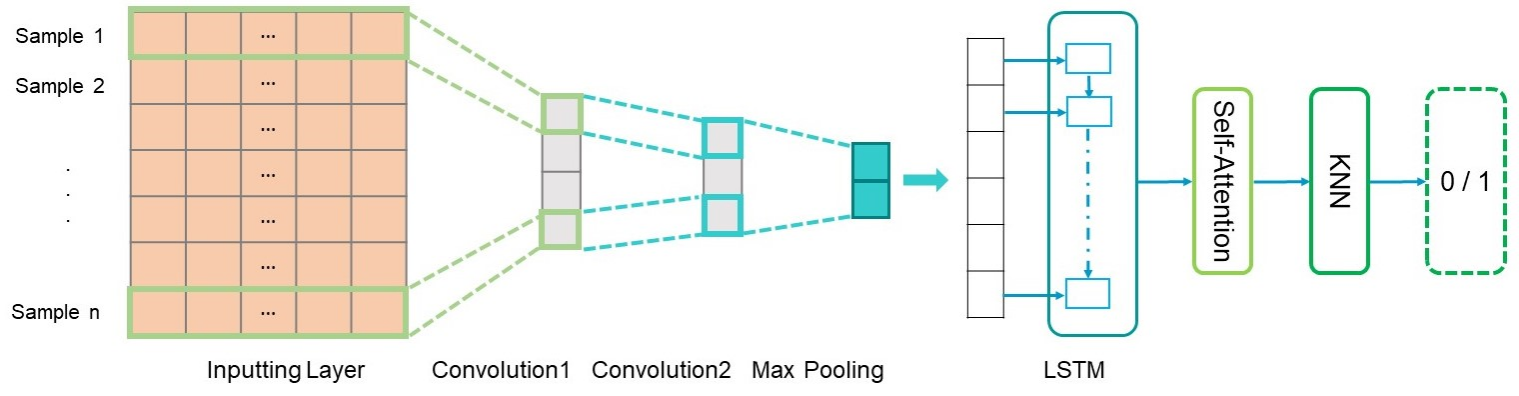
The architecture of our framework.

In this paper, the prediction problem is treated as a classification problem, where a record with a prediction value 1 (0) indicates a positive (negative) instance. Public datasets and a personnel performance prediction dataset were used to train and test the proposed framework. The overview of our framework is illustrated in Figure 1. We propose a CHRNNA framework to achieve our goal. It contains the following steps.

(1) Two kinds of neural networks, CNNs and RNNs, where the latter refers to LSTM, are employed in personnel prediction.This hybrid model combining CNN with LSTM is called the CRNN model;
(2) The self-attention mechanism is introduced to a hybrid CRNN model, which aims to automatically capture the informative features;
(3) The learned features, extracted from the last layer of CRNN model based on self-attention, are directly fed into the KNN classifier as inputs.

Next, we thoroughly describe the data and the two stages contained in the hybrid CRNN model with a self-attention mechanism and classification.

### 3.1. Personnel Performance Prediction Data

#### 3.1.1. Data Description

To further estimate the performance of our method, we used a collected real dataset for a high-technology industry to forecast personnel performance. The characteristics of this dataset are shown in Table 1.

Next, the data collection and data preprocessing process are described in detail.

#### 3.1.2. Data Collection

To be able to employ deep learning for modeling personnel performance, we required a dataset with labeled performance. Because there is no such human-labeled dataset publicly available, we should collect a personnel performance dataset with a performance-carrying label. The experimental dataset is collected from the Human Resource Department of a high-technology industry.

The first issue to consider is what attributes should be collected in this dataset. Salgado proposed the famous Big Five Model, which included five personality factors, i.e., conscientiousness, emotional stability, extraversion, openness, and agreeableness [41]. Güngör et al. thought that determining the most eligible person was dependent on some factors, such as work experience, foreign language, basic computer skill, personal goal, long life learning, etc. [42]. Li et al. pointed out that human and task characteristics are related to a person’s performance. We adopted an expert panel discussion and behavioral event interviews as the main method of collecting data. The panel of experts conducted a content analysis of the interview content to determine the competency characteristics exhibited by the respondents. Many aspects like “self-control”, “confidence”, “initiative”, “self-motivation”, and so on were also identified. After reaching an agreement through discussion, each evaluator was scored. That is, the determination of the attributes values was carried out by the expert group to analyze the content of the interviews. In order to make the collected data satisfy our demands, we created a personnel performance dataset including 5 categories and 22 attributes. These attributes were deemed to have an impact on personnel performance and are shown in detail in Table 1.

The second issue to contemplate is how to describe a person’s performance in this dataset. Just like many decision problems, the personnel performance problem is too sophisticated in real life. Since human behaviors and characteristics are complicated, it is difficult to quantify a person’s performance. People usually forecast inaccurately for quantitative problems, while relatively having an accurate prediction for qualitative problems. Therefore, we used qualitative fuzzy levels to describe attributes.

The components of the dataset are shown in Table 2. It contains 23 items, which includes 22 attributes and 1 personnel performance class. Each feature is represented by 5 fuzzy levels. For instance, a person’s memory capability has been divided into five fuzzy levels, with “very poor”, “poor”, “middle”, “good”, and “very good”. A person’s confidence has five fuzzy levels, which are “very unconfident”, “unconfident”, “medium”, “confident”, and “very confident”. Further, we used five fuzzy levels to describe experience, representing “completely inexperienced”, “inexperienced”, “middle”, “experienced”, and “well experienced”.

For different types of tasks, various means can be used to evaluate the personnel’s performance. Taking a high-technology industry as an example, the performance of personnel is determined by the completion of tasks and the meetings. To facilitate performance prediction by employing a classification algorithm, actual performance values correspond and are transformed to 2 grades, and we used the 2 integers of 0 and 1 to represent different grades of performance in the sample data. That is to say, real performance values are 0 and 1. If personnel performance is achieved, performance value equals to 1, otherwise it equals to 0.

#### 3.1.3. Data Preprocessing

The raw data included some samples that were not applicable. Since these samples would reduce the ability to build a model, we needed to clean the data and remove all the duplicate data. Then, we manually carried out a random inspection of 300 instances from the dataset and found no duplicates. The initial dataset has 1151 samples. After discarding anomalous samples from the dataset, the number of applicable instances was reduced to 1139. The anomalous samples refer to duplicates and the data whose attributes value are all five. In this way, the dataset we attained can utilize deep learning. Thus, we now turn to describing the deep learning method we adopted.

### 3.2. First Stage: A Hybrid CRNN Model with Self-Attention

We call the hybrid model of CNN combined with LSTM as a CRNN model. Next, we describe the details of this hybrid CRNN model with a self-attention mechanism.

#### 3.2.1. CNN for Feature Extraction

A critical problem for personnel performance prediction is feature representation, whereas traditional feature selection methods rely mainly on human-designed features. On one hand, hand-crafted feature extraction would be too time-consuming. On the other hand, hand-crafted feature extraction depends on human experience and requires designers to have strong professional background knowledge. Thus, the classification performance will be affected. Obviously, the disadvantages of this approach are apparent.

Recently, the rapid development of deep neural networks has brought new inspiration to feature extraction. In this section, we used CNNs to model, which we shall now introduce.

Our network uses two convolutional layers and a max-pooling layer, as shown in Figure 1. The convolution layer is used to automatically capture features, and the max-pooling layer is utilized to automatically extract which features play key roles in personnel performance prediction. The first procedure in our model is to train the network by inputting the sample itself and its label. Convolution layers contain a series of feature maps, which are formed by sliding diverse kernels over an input sample. A max-pooling manipulation is employed to capture the most important feature by extracting the biggest value from a feature map. More details about models are described in Section 4.

#### 3.2.2. LSTM for Contextual Information

When people perform tasks, they need some basic qualities, such as perception, learning, creativity, memory, engagement, experience, health, etc. These information sequences are rich in content, and the information has a complex temporal correlation with each other. For example, for creativity, perception, learning, and confidence used in the design process, we need to tackle multidimensional input information at the same time, because it is constantly changing. Moreover, people do not start to carry out their tasks from scratch. As humans do computational design, they perform it based on the previous information. People do not abandon all known information and start to perform tasks from scratch again. However, CNN is unsuitable for modeling temporal information or dependency. LSTM has a memory function, which is extraordinary suitable for addressing this problem.

In this section, for our modeling, we used long short-term memory (LSTM) [43], a variation of RNNs. For a mathematical notation, we denote scalars with an italic lower case (e.g., *h*), vectors with a bold lower case (e.g., **h**), and matrices with a bold upper case (e.g., **U**).

**Figure 2:**
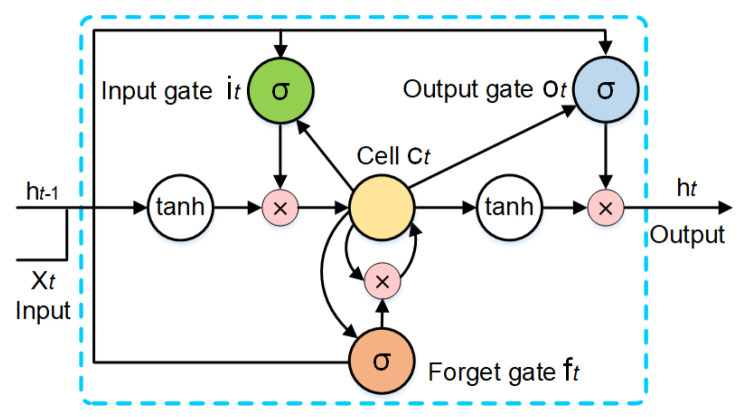
Long short-term memory (LSTM) structure.

LSTM is a kind of neural network architecture that is especially adapted to model sequential information. As illustrated in Figure 2, LSTM includes an input gate, a forget gate, and an output gate. LSTM networks tackle the problem of long-term dependencies of features via enhancing a memory cell at each time step *t*. We used LSTM to model the contextual dependencies and semantic relevance from our datasets, as shown in Figure 1.

LSTM takes an input vector $x_{t}$, a hidden vector $h_{t-1}$, and a memory cell state vector $c_{t-1}$ and generates $c_{t}$ and $h_{t}$ through the following calculations:

(1) $$i_{t}=\sigma \left(W_{i}x_{t}+U_{i}h_{t-1}+b_{i}\right)$$

(2) $$f_{t}=\sigma \left(W_{f}x_{t}+U_{f}h_{t-1}+b_{f}\right)$$

(3) $$o_{t}=\sigma \left(W_{o}x_{t}+U_{o}h_{t-1}+b_{o}\right)$$

(4) $$n_{t}=\tanh\left(W_{n}x_{t}+U_{n}h_{t-1}+b_{n}\right)$$

(5) $$c_{t}=f_{t}⊙c_{t-1}+i_{t}⊙n_{t}$$

(6) $$h_{t}=o_{t}⊙\tanh\left(c_{t}\right)$$

where $i_{t}$, $f_{t}$, $o_{t}$, and $c_{t}$ are the input gates, forget gates, output gates, and memory cell, respectively. The $n_{t}$ represents a new memory cell vector with candidates which could be added to the state. σ(·) is logistic sigmoid function, and tanh(·) is hyperbolic tangent function. ⊙ refers to the element-wise multiplication operation. The LSTM parameters **W**, **U** are weights, and **b** is bias, where $W_{k}$, $U_{k}$, and $b_{k}$ are for k∈{i,f,o,n}.

#### 3.2.3. Self-Attention

By combining convolutional layers, max-pooling layer, and recurrent neural networks, our model adopts the strength of both convolutional neural models and recurrent neural models. Moreover, we want to capture long-range dependencies from personnel performance information. Recently, a self-attention mechanism has led to new ideas for solving these problems.

We think that 5 categories and 22 attributes have impacts on personnel performance in this paper. However, different features have various importance. Therefore, we require a strategy to discriminate the importance of the 22 attributes. To forecast personnel performance, the self-attention mechanism has the ability to identify the significance of diverse attributes rather than considering each attribute on average. Thus, we used the self-attention mechanism for the hybrid CRNN model.

In this section, we thoroughly describe self-attention. After obtaining the contextual features of the input sample, the self-attention mechanism [44] is used to learn the weight coefficient, which reflects the importance of each feature in the sample. Suppose that for each instance, we have N series of output from N LSTM cells, then the self-attention can be formulated as:

(7) $$c_{i}=\sum_{j=1}^{N}\alpha_{ij}v_{i}$$

We compute the weight coefficient $\alpha_{ij}$ of each $v_{i}$ according to the following formulation:

(8) $$\alpha_{ij}=\frac{exp(v_{ij})}{\sum_{k=1}^{N}exp\left(v_{ik}\right)}$$

(9) $$v_{ij}=Fscore\left(v_{i},v_{j}\right)$$

where $c_{i}$ is the representation of the sequence as the weighted sum of hidden representation, $\alpha_{ij}$ is the normalized importance, $v_{ij}$ indicates the score about the degree of dependency between $v_{i}$, and $v_{j}$, Fscore is a function to compute the score about $v_{i}$ and $v_{j}$. $v_{ij}$ calculated by function F-score is normalized by softmax via Equation (8). The output of this self-attention mechanism is a weighted sum.

Self-attention is seen as a separate layer, mixed with the CNN and LSTM model, which is able to more fully integrate their respective strengths.

#### 3.2.4. Model Training

In recent years, the cross-entropy loss function has been widely used as a loss function in the model training of various tasks. Cross-entropy refers to the gap between the true probability distribution and the predicted probability distribution. In model training processes, we expect the probability that the model predicts the instance to be as similar as possible to the true probability. The formulation is defined as:

(10) $$CE\left(p,y\right)=-\sum_{i=1}^{N}y_{i}log\left(P\right)$$

where *i* denotes the sample, *N* represents the total number of the samples, *y* is the one-hot vector corresponding to the true category of the sample, and *P* is the predicted probability. In this study, we used cross-entropy as a loss function. We expect the cross-entropy loss function to be minimized, because the lower the cross-entropy is, the closer the prediction distribution obtained by our model is to the true distribution.

### 3.3. Second Stage: Classification

The previous stage (neural network training) can be viewed as preprocessing because it can be executed independently before the classification stage.

At present, our society is besieged with large-scale data, and there is usually no prior knowledge of data distribution. The KNN classifier is very suitable for addressing these data. Further, KNN can get more complex decision information than the activation function of the last layer of our hybrid CRNN model. The idea of proposing our method is that these processes can complement each other perfectly and obtain the synergy of large datasets.

When it comes to classifying samples that need to be classified, our solution is described below:

(1) The raw instances are propagated via our proposed network, and their feature vectors are extracted from the last layer of the hybrid CRNN model with self-attention;
(2) The learned features mentioned above are fed into the KNN classifier as inputs;
(3) The distances of the samples are computed, and the nearest training samples belonging to the test samples are selected;
(4) The conventional KNN classification is carried out within these chosen data.

Next, we did extensive experiments on a personnel performance dataset to validate the goodness of our approach. More details about experiments are given in Section 4.

## 4. Experiments

In this section, our experiment and discussion are detailed. It includes the experimental setup, comparison algorithms, evaluation criterion, various experimental results to evaluate the performance of the presented scheme, and analysis of the case study.

### 4.1. Experimental Setup

This subsection introduces our experimental settings in detail. All experiments were performed on Python programming language (version 3.5), employing TensorFlow (version 1.10) and Keras (version 2.2). The PC we used includes an Intel(R) Core(TM) i5-4570 CPU running at 3.20 GHz. For a higher computing performance, we used a NVIDIA GeForce GTX 750 GPU with the cuDNN library.

In this experiment, the dataset was divided into the training set (60%), test set (20%), and validation set (20%). The learning of the CHRNN (without a self-attention mechanism) and CHRNNA framework (with a self-attention mechanism) was carried out for 200 epochs and a mini-batch size of 128 samples. Layer 1 with a convolution operator has 13 filters of size 1 × 2; Layer 2 with a convolution operator contains 26 filters of size 1 × 2, followed by a max-pooling layer. The max-pooling layer has a kernel size 2 and stride step 1. The dimension of the LSTM layer is 64. The dropout rate was set to 0.5 to prevent overfitting. For the binary classification problem, we adopted the sigmoid activation function. We employed binary cross-entropy loss on sigmoid function and the Adam optimization method, which works better to avoid the gradient vanishing/exploding issues. In the final classification stage, we studied different values of the number of neighbors k. More precisely, the k value considered is 7. Table 3 lists our hyper-parameter setting.

### 4.2. Comparison Algorithms

To demonstrate that our model is an excellent model, we introduced several comparison algorithms. We selected some traditional models, such as C4.5, RIPPER (repeated incremental pruning to produce error reduction), CBA (classification based on associations), CMAR (classification based on multiple association rules), MKNN (modified KNN) [45] and EEKNN (entropy Euclidean distance KNN). In addition, we also selected some deep learning models (e.g., CNN, RNN, and LSTM) as comparison methods of personnel performance prediction. We considered these traditional models and deep learning models as baselines.

- **C4.5** is a popular and powerful decision tree classifier;
- **RIPPER** is a traditional rule-based classifier, whose accuracy may not be as high in most cases;
- **CBA** is an important association rule-based classifier, which generates all the association rules with certain support and confidence thresholds;
- **CMAR** is an association rule-based classifier, which uses multiple rules for prediction;
- **MKNN** is an approach based on local learning which first introduces a new similarity function;
- **EEKNN** is a performance modeling method based on data mining. A proposed improved KNN algorithm was used to handle the personnel performance prediction problem;
- **CNN** is a popular model which is used for feature learning;
- **RNN** is a deep learning model for sequential data modeling. Here, we considered it as a comparison method;
- **LSTM** is a variant of RNN. It is an advanced RNN architecture which prevents vanishing gradients or the phenomena of exploding;
- **CHRNN** is our method. It is a general framework without a self-attention mechanism;
- **CHRNNA** is our method. It is our final selected framework which integrates the self-attention mechanism.

### 4.3. Evaluation Criterion

To quantitatively evaluate the classification performance of the proposed framework, we took the accuracy (acc) as a performance measure. We used the acc to compare the classification performance of different algorithms. The index acc is defined by true positive (TP), false negative (FN), false positive (FP), and true negative (TN), which is computed based on Equation (11):

(11) $$\mathrm{acc}=\frac{\mathrm{TP}\;+\;\mathrm{FN}}{\mathrm{TP}\;+\;\mathrm{FN}\;+\;\mathrm{FP}\;+\;\mathrm{TN}}$$

The index acc is in the range of [0,1]. The bigger the value of acc is, the better the performance of the classifier.

Moreover, we also used precision (P), recall (R), and the F1 value as evaluation metrics to evaluate the performance of our proposed methods in this paper. The formula for computing P, R, and F1 is as follows:

(12) $$P=\frac{\mathrm{TP}}{\mathrm{TP}\;+\;\mathrm{FP}}$$

(13) $$R=\frac{\mathrm{TP}}{\mathrm{TP}\;+\;\mathrm{FN}}$$

(14) $$F1=\frac{2\times P\times R}{P\;+\;R}$$

### 4.4. Experimental Results

#### 4.4.1. Experimental Results on a Personnel Performance Prediction Dataset

**Figure 3:**
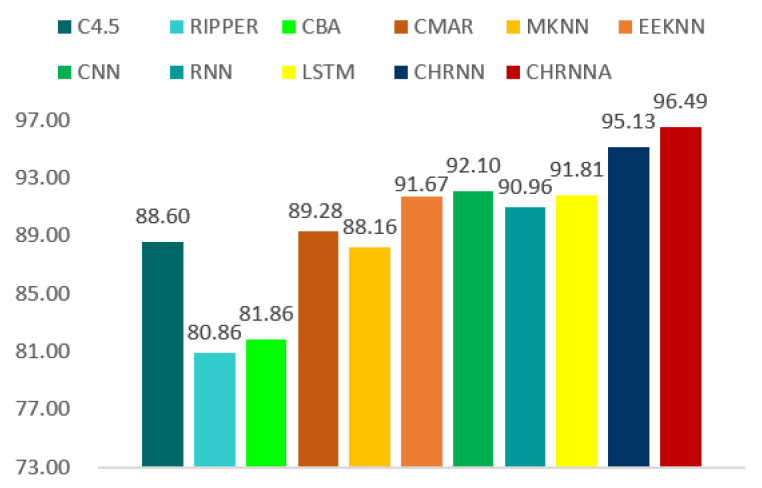
Performance of all the methods on personnel performance prediction.

To further demonstrate the effectiveness and applicability of the proposed method, we introduced several methods for comparison on a real personnel performance prediction dataset. We repeated our experiments 10 times and reported the average results using the acc metric, as shown in Figure 3. We collected data from a high-technology industry. All samples were divided into two categories—one achieved personnel performance, the other did not achieve personnel performance. The forecasted values were compared with the true values to validate the applicability of our framework.

The previous work is based on traditional machine learning methods, while our approaches used deep learning. This paper compares the methods of C4.5, RIPPER, CBA, CMAR, MKNN, EEKNN, CNN, RNN, and LSTM. Figure 3 shows the performance comparisons. Our methods of CHRNN and CHRNNA achieved 95.13% and 96.49%, respectively. For the traditional machine learning method, EEKNN achieved an accuracy of 86.84%. In comparison with the baseline scheme EEKNN, CHRNN generated up to 8.29% accuracy improvement. Moreover, thanks to the introduction of the self-attention strategy, our CHRNNA method generated up to 1.36% accuracy increment again. For deep learning methods, CNN and LSTM achieved an accuracy of 92.10% and 91.81%. Compared to CNN and LSTM, our CHRNNA increased by 4.39% and 4.68%. The best performance was achieved by CHRNNA. When we compare deep learning methods (e.g., CHRNN and CHRNNA) to the traditional method (e.g., EEKNN) and existing deep learning methods (e.g., CNN and LSTM), the experimental results show that our deep learning methods outperform other methods for personnel performance prediction.

As Table 4 shows, we also adopted P, R, and F1 score to assess the performance of our method. The P, R, and F1 of CHRNNA on personnel performance data were 97.07%, 96.14%, and 96.60%, respectively, which are all higher than existing methods. The higher metrics values of the CHRNNA framework show CHRNNA has a better performance. It shows that the CHRNNA framework is more reliable when applied to personnel performance prediction.

From Figure 3 and Table 4, we conclude that the CHRNNA framework generally performs better than other methods. The reasons behind these phenomena are as follows:

Firstly, for C4.5, it only produces a small set of rules which may result in many interesting and useful rules not being discovered. For CBA and CMAR, they generate a very large number of potential classification rules. These approaches do not easily identify the most effective rule for classifying a new case. They simply select a rule with a user-defined measure, such as confidence. Such a simple pick may affect the classification performance, and their confidence-based rule evaluation measure may lead to overfitting. The results of machine-learning-based methods are also not encouraging. However, our method is different. Our CRNN model can automatically learn discriminative features, which avoids inefficient and cumbersome process. The CRNN model applies a recurrent structure to better learn contextual information of features. When humans carry out tasks, they do not start from scratch. They need to deal with multiple input information of a high dimension at the same time, which has temporal correlation. CRNN has a memory function that is suitable for solving the problems of temporal information or dependence in personnel performance prediction. Our model uses the advantage of both convolutional neural models and recurrent neural models.

Secondly, KNN can attain more complex decision information than the activation function in the last layer of the hybrid CRNN model. This fact clearly shows the advantage of the presented combined method of the CRNN model and KNN algorithm compared to conventional machine learning methods. It is interesting that CRNN and KNN reinforce one another, which achieves a better classification performance.

Finally, we summarize that CHRNNA is superior over the general CHRNN framework. The application of our approach depends on the size and attribute diversity of the instances. Different features have different effects on personnel performance. We used self-attention strategy to discriminate the importance of various features. The most important features are the most prioritized information in the self-attention layer. We believe that the self-attention layer has the ability to learn long dependencies without considering the distance in sequences. The self-attention mechanism is helpful to achieve a high-quality prediction in accuracy.

In summary, we are content to find that deep learning techniques can help in improving the performance. To be precise, the self-attention mechanism can further improve the classification performance. We believe that our proposed method can be successfully applied to personnel performance prediction.

#### 4.4.2. The Time Efficiency Analysis

Computation time is another way to compare different methods. The running time of these algorithms is shown in Table 5.

The running time of the EEKNN algorithm took 52.04 s, while CHRNNA was almost six times faster, 8.89 s. The total running time of the MKNN algorithm is the lowest. The running time in seconds shows that the CHRNNA method is significantly faster than the MKNN and EEKNN algorithms. Although our proposed method is somewhat slower than several methods in running time, it has a significant performance improvement in terms of acc, P, R, and F1 evaluation indices. With the continuous improvement of computing power, it can make up for the lack of response time. Thus, the difference in running time is negligible. Combining comparison results in terms of acc, P, R, F1, and running time, we can conclude that the CHRNNA method is more effective than the compared algorithms.

### 4.5. Ablation Study of Various Modules

To further evaluate the effectiveness of various modules, we reported the ablation study of our CHRNNA model. Our main model outperforms the other variants. We applied our CHRNNA model and its two submodels, which are without self-attention and LSTM. The experimental results for the ablation study are listed in Table 6. The results of the “- self-attention” row refer to the results without the self-attention mechanism. The results of the “- LSTM” row refer to the results without LSTM. The results indicate that: (1) our main model is better than the two variants under the same settings; and (2) as expected, the results for the simplified models all drop a lot. This clearly demonstrates the effectiveness of these modules. For the submodel “- self-attention”, the drop of ACC, P, R, and F1 demonstrates that the self-attention mechanism is critical. Specifically, the self-attention mechanism helps to evaluate the contribution of various attributes, so we can identify the most influential factor for the final performance decision. The drop in the performance of “- LSTM” also shows that LSTM is important for achieving a good performance.

## 5. Conclusions

High-technology industries depend on personnel performance to maintin their competitive advantages. In this paper, we presented a novel CHRNNA framework, which is used to forecast personnel performance in the future and help decision-makers to select the most adequate talents. We designed and collected a dataset with 22 attribute items, which were used to reflect personnel performance.

The proposed framework remarkably improves prediction performance. In the first stage, we employ a hybrid CRNN model with a self-attention mechanism to automatically capture the global informative features. In the second stage, we use a KNN-based classifier to forecast personnel future performance. Experimental results demonstrate that our proposed method yields a significant performance.

In our opinion, there is some room for further improvement with minimal accuracy losses, such as studying more advanced network architectures and designing a loss function, which will be the focus of our future work. 

## Figures and Tables

**Table 1:** The personnel performance dataset used for experimentation.

Datasets	Instances	Attributes	Classes
Personnel Performance	1139	22	2

**Table 2:** The components of the personnel performance dataset.

Category	Attributes	Value	Description
Capability	Perception	1–5	5 levels of perception
	Learning	1–5	5 levels of learning
	Memory	1–5	5 levels of memory
	Creativity	1–5	5 levels of creativity
	Speech	1–5	5 levels of speech
	Logic	1–5	5 levels of logic
Conscientiousness	Dedication	1–5	5 levels of dedication
	Engagement	1–5	5 levels of engagement
	Self control	1–5	5 levels of self control
	Decision making	1–5	5 levels of decision making
Achievement	Goal	1–5	5 levels of goal
	Initiative	1–5	5 levels of initiative
	Independence	1–5	5 levels of independence
	Confidence	1–5	5 levels of confidence
Emotion	Emotion awareness	1–5	5 levels of emotion awareness
	Emotion expression	1–5	5 levels of emotion expression
	Self-motivation	1–5	5 levels of self-motivation
	Emotion management	1–5	5 levels of emotion management
Complementary quality	Education	1–5	5 levels of education
	Health	1–5	5 levels of health
	Experience	1–5	5 levels of experience
	Stress tolerance	1–5	5 levels of stress tolerance
Personnel performance		0, 1	0, not achieve performance; 1, achieve performance

**Table 3:** Parameter settings.

Parameters	Parameters Value
epochs	200
batch size	128
convolution 1 layer: filters number	13
convolution 2 layer: filters number	26
filters size	1 × 2
LSTM dimension	64
Dropout rate	0.5

**Table 4:** Performance of comparison methods on personnel performance data in terms of precision (P), recall (R), and F1 (%). The best experimental results are given in bold.

Metrics	C4.5	RIPPER	CBA	CMAR	MKNN	EEKNN	CNN	RNN	LSTM	Ours	
										CHRNN	CHRNNA
P	71.47	82.52	80.86	57.16	67.22	71.93	92.14	89.41	92.06	96.02	**97.07**
R	78.92	85.01	88.36	79.28	60.54	67.12	92.54	89.04	91.67	94.61	**96.14**
F1	74.32	83.70	84.11	60.02	62.68	69.12	92.34	89.22	91.86	95.31	**96.60**

**Table 5:** Computation time of contrastive experiments measured in seconds per run.

C4.5	RIPPER	CBA	CMAR	MKNN	EEKNN	CNN	RNN	LSTM	CHRNN	CHRNNA
0.82	5.44	4.52	11.31	1878.56	52.04	5.07	3.27	6.56	12.29	8.89

**Table 6:** Ablation study of CHRNNA with various modules on personnel performance. The best results are given in bold.

Strategy	ACC	Δ1	P	Δ2	R	Δ3	F1	Δ4
CHRNNA (Our Model)	**96.49**	-	**97.07**	-	**96.14**	-	**96.60**	-
- Self-attention	95.13	−1.36	96.02	−1.05	94.61	−1.53	95.31	−1.29
- LSTM	95.83	−0.66	96.04	−1.03	95.61	−0.53	95.81	−0.79
